# Diversity of Microsporidia, *Cryptosporidium* and *Giardia* in Mountain Gorillas (*Gorilla beringei beringei*) in Volcanoes National Park, Rwanda

**DOI:** 10.1371/journal.pone.0109751

**Published:** 2014-11-11

**Authors:** Bohumil Sak, Klára J. Petrželková, Dana Květoňová, Anna Mynářová, Kateřina Pomajbíková, David Modrý, Michael R. Cranfield, Antoine Mudakikwa, Martin Kváč

**Affiliations:** 1 Institute of Parasitology, Biology Centre of the Academy of Sciences of the Czech Republic, v.v.i., České Budějovice, Czech Republic; 2 Institute of Vertebrate Biology, Academy of Sciences of the Czech Republic, v.v.i., Brno, Czech Republic; 3 Liberec Zoo, Liberec, Czech Republic; 4 Department of Pathology and Parasitology, Faculty of Veterinary Medicine, University of Veterinary and Pharmaceutical Sciences, Brno, Czech Republic; 5 Faculty of Science, University of South Bohemia in České Budějovice, České Budějovice, Czech Republic; 6 CEITEC - Central European Institute of Technology, University of Veterinary and Pharmaceutical Sciences, Brno, Czech Republic; 7 Gorilla Doctors, Karen C Drayer Wildlife Health Center, Davis, CA, United States of America; 8 Rwanda Development Board (RDB), Kigali, Rwanda; 9 Faculty of Agriculture, University of South Bohemia in České Budějovice, České Budějovice, Czech Republic; Cornell University, United States of America

## Abstract

**Background:**

Infectious diseases represent the greatest threats to endangered species, and transmission from humans to wildlife under increased anthropogenic pressure has been always stated as a major risk of habituation.

**Aims:**

To evaluate the impact of close contact with humans on the occurrence of potentially zoonotic protists in great apes, one hundred mountain gorillas (*Gorilla beringei beringei*) from seven groups habituated either for tourism or for research in Volcanoes National Park, Rwanda were screened for the presence of microsporidia, *Cryptosporidium* spp. and *Giardia* spp. using molecular diagnostics.

**Results:**

The most frequently detected parasites were *Enterocytozoon bieneusi* found in 18 samples (including genotype EbpA, D, C, gorilla 2 and five novel genotypes gorilla 4–8) and *Encephalitozoon cuniculi* with genotype II being more prevalent (10 cases) compared to genotype I (1 case). *Cryptosporidium muris* (2 cases) and *C. meleagridis* (2 cases) were documented in great apes for the first time. *Cryptosporidium* sp. infections were identified only in research groups and occurrence of *E. cuniculi* in research groups was significantly higher in comparison to tourist groups. No difference in prevalence of *E. bieneusi* was observed between research and tourist groups.

**Conclusion:**

Although our data showed the presence and diversity of important opportunistic protists in Volcanoes gorillas, the source and the routes of the circulation remain unknown. Repeated individual sampling, broad sampling of other hosts sharing the habitat with gorillas and quantification of studied protists would be necessary to acquire more complex data.

## Introduction

Microsporidia, *Cryptosporidium* spp. and *Giardia* spp. are unicellular parasites spread by the fecal-oral route by environmentally resistant stages and they can infect humans, livestock and wildlife animals including non-human primates [1]–[5]. The symptoms in immunocompetent hosts are usually mild and self-limiting. However, human cryptosporidiosis, microsporidiosis and giardiosis emerged as important opportunistic diseases when AIDS became pandemic [6], [7]. Thousands of HIV-infected patients with chronic diarrhea attributed to these organisms have been reported from all over the word [7], [8].

Recent studies surprisingly showed high prevalence of asymptomatic microsporidial infection in captive and habituated great apes [3], [5], while *Cryptosporidium* and *Giardia* spp. seems to be less frequent pathogen [1], [3], [9]. It has been suggested, that increased prevalence of these pathogens could be a result of frequent presence of humans and livestock in the ranges of primates [4], [9]–[11], rather than resulting from close contact with humans [12]. Moreover, as opportunistic pathogens they are not strictly host specific and hence able to cross the species transmission barrier [13]–[16]. However, only genotyping and subtyping of particular isolates can provide the essential information about epidemiology and zoonotic potential of these protists in primates [11], [17]–[19].

The mountain gorilla (*Gorilla beringei beringei*) is classified as an endangered species with only two isolated populations remaining in Bwindi Impenetrable National Park, Uganda (330 km^2^) and the Virunga Massif (450 km^2^) at the borders of Rwanda, Uganda, and the Democratic Republic of Congo [20], [21]. Observing habituated mountain gorillas has proven particularly popular since it began in the 1950s, and is one of the world’s best-known wildlife experiences [22]. During the habituation process animals become accustomed to human presence and are thought eventually to accept a human observer as a neutral element in their environment [23]. However, the consequences of increasing human-gorilla contacts in habituated groups can have negative effects on animals [24]–[29]. An increased anthropogenic impact on primate populations may result in general changes in communities of their parasites, and also in a direct exchange of parasites between humans and primates [30], [31].

Since only limited work has been done to explore the molecular diversity of gastrointestinal parasites in mountain gorillas [1], [2], [32]–[34] we conducted a comprehensive molecular screening for *Encephalitozoon* spp., *Enterocytozoon bieneusi*, *Cryptosporidium* spp. and *Giardia* spp. in several groups of habituated mountain gorillas (*Gorilla beringei beringei*) in Volcanoes National Park, Rwanda.

## Materials and Methods

### Ethics Statement

The research complied with the legal requirements of Rwanda and adhered to the research protocol submitted to conservation authorities. The permission to collect the fecal samples was obtained from Rwanda's Office of Tourism and National Parks. Since the collection of fecal samples from gorillas was noninvasive and did not cause any observable distress to the animals, no animal ethic committee was consulted regarding our study. The sampling was performed during routine health checks by Mountain Gorilla Veterinary Project and Dian Fossey Gorilla Fund employees. No interaction with animals was conducted for this study.

### Study site

The study was conducted in Volcanoes National Park in North western Rwanda, a montane rain forest that ranges in altitude from 2300 to 4500 m and supports distinct vegetation communities at different elevations [35], [36]. The area is surrounded by some of the highest rural human population densities in the world, up to 820 people per km^2^ [37]. High densities of humans can have negative impact on conservation of wildlife in the Park [38]. Virunga subpopulation of mountain gorillas have suffered numerous threats such as habitat destruction for firewood and agriculture, illegal cattle grazing, illegal logging and illegal poaching [39]. Although *G. beringei beringei* was affected by war and instability, currently the park has undergone through significant regeneration, with investment into tourism, improvement of social infrastructure and safety [40], [41].

### Studied gorilla groups and sample collection

In September 2007, we collected 100 individual fecal samples from night nests of seven gorilla groups that were habituated and used either for tourism (5) or for research (2) (Table 1). Usually four trackers and up to four researchers visit the research groups for approximately four hours every day. The tourist groups are visited by four local trackers, two local guides and up to eight tourists for an hour daily at high booking seasons. People following both tourist and research groups are accompanied by military men and porters who stay approximately 100 yards away from gorillas for the safety reasons. In 2007, Volcanoes NP Rwanda has reported 18,000 tourists visiting the Park [42].

**Table 1:** *Enterocytozoon bieneusi, Encephalitozoon cuniculi, Cryptosporidium* spp. and *Giardia* spp. infection in mountain gorillas (*Gorilla beringei beringei*).

Groups	n	Positive samples			
		*Encephalitozoon* spp.	*Enterocytozoon bieneusi*	*Cryptosporidium* spp.	*Giardia* spp.
PABLO^1^	20	4×ECII	1×gorilla 2, 1×gorilla 4, 1×EpbA	2×*C. meleagridis*	-
SHINDA^1^	18	5×ECII	1×gorilla 7, 1×gorilla 8, 1×C, 1× D	2×*C. muris*	-
AMAHORO^2^	11	-	1×EpbA	-	-
UMUBANO^2^	6	-	2×EpbA	-	-
SABINYO^2^	8	1×ECI	1×EpbA	-	-
SUSA^2^	21	1×ECII	1×gorilla 5, 1×gorilla 6, 1×C, 1×EpbA	-	-
KWITONDA^2^	16	-	2×D, 1×EpbA	-	-
	**100**	**11**	**18**	**4**	**0**

1 gorilla group habituated for research;

2 gorilla group habituated for tourism; **D** = *E. bieneusi* genotype D; **gorilla 1** = *E. bieneusi* genotype gorilla 1; **gorilla 2** = *E. bieneusi* genotype gorilla 3; **gorilla 3** = *E. bieneusi* genotype gorilla 3; **EC I** = *E. cuniculi* genotype I; **EC II** = *E. cuniculi* genotype II; **n** number of samples.

To prevent the repeated sampling of same individuals, which could distort the parasite prevalence estimation, each group was sampled only once. All fecal samples were immediately preserved in 96% ethanol and transported to the Institute of Parasitology, Biology Centre of Academy of Sciences of the Czech Republic.

### DNA extraction, PCR amplification, sequencing and genotyping

The suspension of each fecal sample in alcohol was evaporated overnight at 60°C. A total of 200 mg of fecal samples were homogenized by bead disruption using 0.5 mm glass beads (Biospec Products, Inc., Bartlesville, OK, USA) in a FastPrep-24 Instrument (MP Biomedicals, Santa Ana, CA, USA) at a speed of 5 m/s for 1 min followed by isolation/purification using the QIAamp DNA Stool Mini Kit in accordance with the manufacturer’s instructions (Qiagen, Hilden, Germany). Purified DNA was stored at −20°C prior to use in PCR. All DNA samples obtained for the study were analyzed by polymerase chain reaction (PCR) using sets of specific primers. A nested PCR approach was used to amplify a region of the internal transcribed spacer (ITS) of *Enterocytozoon bieneusi* (∼390 bp) [43], the small ribosomal subunit rRNA (SSU) gene of *Cryptosporidium* spp. (∼830 bp) [44], and the triosephosphate isomerase (TPI) gene of *Giardia intestinalis* (also called *Giardia lamblia*, *Giardia duodenalis*) (∼500 bp) [45]. The following primers sets were used to amplify *Encephalitozoon* spp.: the int580f and int580r primer set for primary PCR analysis [46] and the MSP3 and MSP4 primer set for secondary PCR (∼320 bp) [47]. Secondary PCR products were run on a 2% agarose gel containing 0.2 µg/ml ethidium bromide in 1× TAE buffer at 75 volts for approximately 1 hour. Bands of the predicted size were visualized using a UV light source, cut from the gel, and then extracted using QIAquick Gel Extraction Kit (Qiagen, Hilden, Germany). Gel purified secondary products were sequenced in both directions with an ABI 3130 genetic analyzer (Applied Biosystems, Foster City, CA, USA) using the secondary PCR primers and the BigDye1 Terminator V3.1 cycle sequencing kit (Applied Biosystems, Foster City, CA, USA) in 20 µl reactions.

Positive and negative controls were included in each analysis. DNA from *E. intestinalis* spores grown *in vitro* in the Laboratory of Veterinary and Medical Protistology at the Institute of Parasitology of ASCR, from *E. bieneusi* spores of genotype S6 originally isolated from a eastern house mice, from *Cryptosporidium serpentis* originated from corn snake, and from *Giardia intestinalis* assemblage E originated from the domestic goat were used as positive control for appropriate PCR. All samples were analyzed in duplicate. In case of positive detection, the sample was newly re-isolated and previous finding was independently verified.

### Phylogenetic analyses

The nucleotide sequences of each gene obtained in this study were edited using the software ChromasPro 1.5 (Technelysium, Pty, Ltd., Brisbane, Australia) and were aligned with each other and with reference sequences from GenBank using ClustalX 2.0.6. Alignment adjustments were made manually to remove artificial gaps using BioEdit. Phylogenetic analyses were performed using the software MEGA5 [48]. Neighbour joining (NJ) trees were constructed. All ambiguous positions were removed for each sequence pair. The reliability of branches in trees was assessed using the bootstrap analysis with 1000 pseudo-replicates, with values above 50% reported. Phylograms were drawn using the MEGA5 and were manually adjusted using CorelDrawX5 (Ottawa, Canada). ITS and SSU sequences have been deposited in GenBank under the accession numbers KJ469967–KJ469979, respectively.

### Statistical analyses

To analyze the differences in microsporidia (*Enterocytozoon* spp, *Encephalitozoon* spp.) infections between groups habituated for tourism and research we fitted the generalized linear mixed model (GLMM) with binomial distributions. Samples were classified according to the “group” (Pablo, Shinda, Amahoro, Umubano, Sabinyo, Susa, Kwitonda) and “habituation” (research, tourist). The random factor “group” was nested into the fixed factor “habituation”. Statistical analyses were conducted in R 2.13.1. [49].

## Results

Out of the total of 100 examined gorilla samples 33 were positive for tested parasites. The most frequently detected parasites were *E. bieneusi* in 18 and *Encephalitozoon cuniculi* in 11 samples. *Cryptosporidium* spp. was identified in 4 samples and *Giardia* spp. in none of them (Table 1).

**Figure 1:**
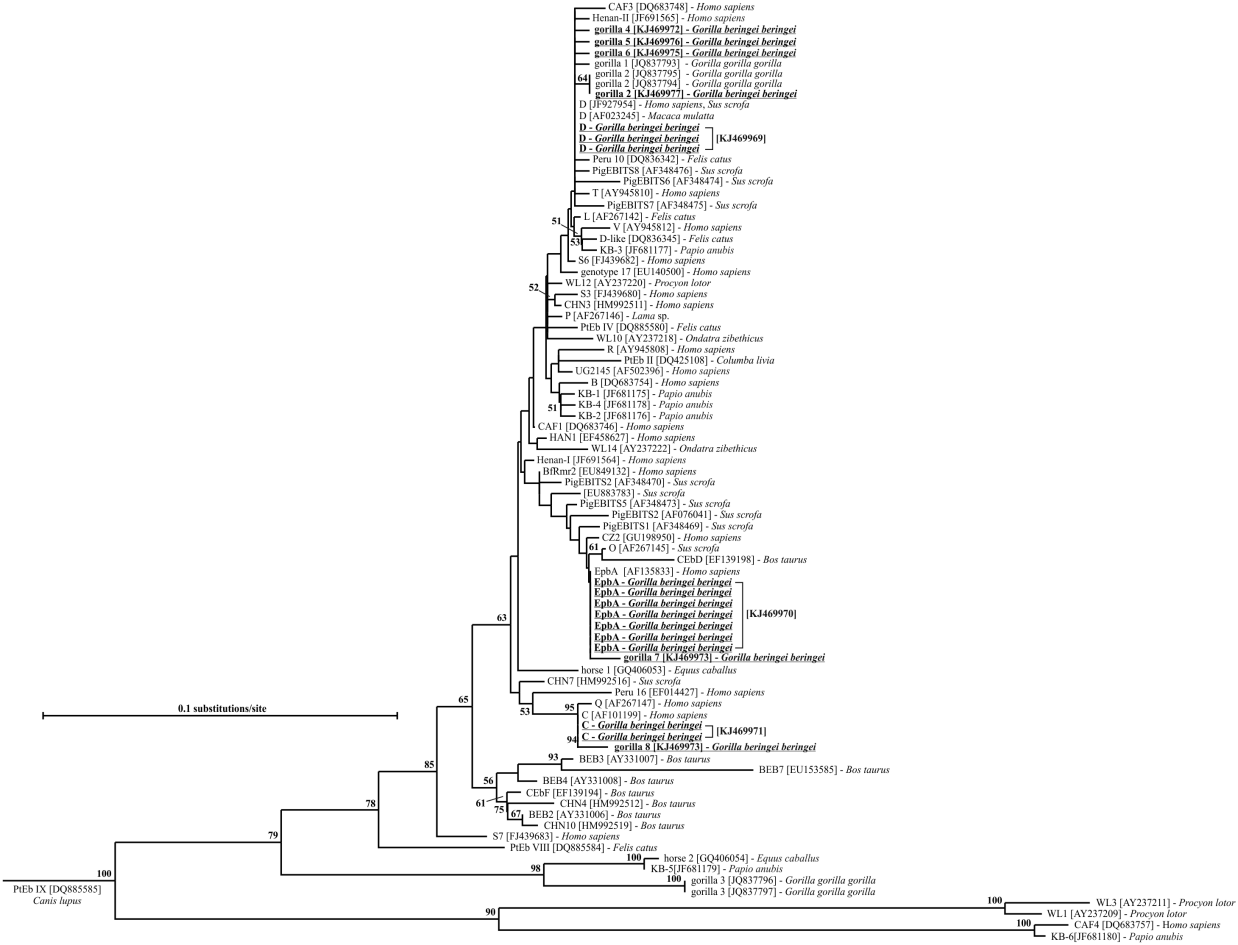
Neighbor-Joining tree based on nucleotide sequences of whole ITS region of *Enterocytozoon bieneusi* isolates, including our new sequences (underlined). The host is listed for each sample. Values on branches are percent bootstrapping using 1 000 replicates. The bootstrap proportions greater than 50% are shown at each branch. Nucleotide sequences generated from this study are underlined and are deposited in the GenBank under Accession Numbers KJ469967–KJ469979.

The alignment of the obtained microsporidial ITS sequences with reference sequences showed 100% homology with GenBank-listed species and their genotypes as follows: 10 *E. cuniculi* genotype II (GQ422153) and 1 *E. cuniculi* genotype I (AF338410) (Table 1). A phylogenetic analysis of all ITS sequences performed on a multiple alignment that included representatives of *E. bieneusi* genotypes accessible in GenBank revealed the presence of sequences matching with previously described human pathogenic *E. bieneusi* genotype D (JF927954) in three cases, seven EpbA (AF135833) and two *E. bieneusi* genotype C (AF101199). Moreover, one genotype previously described from western lowland gorillas, gorilla 2 (JQ837794) was identified in one animal. Furthermore, five sequences of our isolates belonged to five novel genotypes, named gorilla 4–8. While two new genotypes gorilla 7 and gorilla 8 clustered closely to genotype EpbA and C, respectively, genotypes gorilla 4–6 formed a group with isolates from humans and pigs. The global topology of the tree is shown in Fig. 1.

Phylogenetic analyses based on SSU sequences showed that *Cryptosporidium* originating from the Pablo group were 100% similar to *C. meleagridis* (AF112574) and those from Shinda group to *C. muris* (AB089284) (Table 1).

Single-species infection was detected in most animals with the exception of three individuals co-infected with *C. meleagridis* and *E. cuniculi* genotype II, *C muris* and *E. bieneusi* genotype D and *E. cuniculi* genotype II and *E. bieneusi* genotype gorilla 2, respectively (Table 1).

*Cryptosporidium* infections were found only in animals from research groups. We did not detect any differences in the occurrence of *Enterocytozoon bienusi* between research and tourist groups (GLMM, treatment contrasts given: z = –0.086 p = 0.932), but the *Encephalitozoon cuniculi* was more prevalent in research groups compared to tourist ones (GLMM, treatment contrasts given: z = –2.742, p = 0.006). However, the four of five novel genotypes of *E. bieneusi* were found in research groups (Table 1).

## Discussion

To our knowledge, this is the first study providing detailed molecular information about *Cryptosporidium* spp., *Encephalitozoon cuniculi* and *Enterocytozoon bieneusi* infections in wild mountain gorillas in the Virunga Volcanoes region.

While our results show that microsporidia, especially various *E. bieneusi* genotypes reached a prevalence of 29%, results of previous studies revealed that 11% of gorilla samples from Bwindi NP contained *Cryptosporidium parvum* compared to *E. intestinalis* in 3% and *G. intestinalis* in 2% of samples [1]–[3], [33]. On the contrary, *E. cuniculi* was detected as predominant species among western lowland gorillas in the Dzanga-Sangha Protected Areas in Central African Republic [12].

We did not detect any species of protists previously identified in mountain gorillas, namely *Giardia intestinalis*, *Cryptosporidium parvum* and *Encephalitozoon intestinalis* [1]–[3], [32]–[34]. Although most of microsporidia species detected in our study (with exception of the novel genotypes) were already detected in western lowland gorillas [12], *C. muris* and *C. meleagridis* were identified in gorillas for the first time.

We detected 9 different genotypes including *E. bieneusi* genotype EbpA, D, and C described from humans, non-human primates from zoo’s and domestic animals including pigs and cattle [5], [50]–[54]. In previous study only four distinct genotypes of *E. bieneusi*, including genotype D and gorilla 1–3, have been described from western lowland gorillas with genotype D as predominant [12].

All five new *E. bieneusi* genotypes from this study (gorilla 4–8), together with genotype gorilla 2 reported in western lowland gorillas, were genetically related to genotype D and belonged to group 1, as identified by Thellier and Breton [55]. Genotypes of this group are known to have zoonotic potential and they are of public health significance [55]. However, the potential role of humans and domestic animals as a source of infection for mountain gorillas remains speculative, since no current data about microsporidia in hosts sharing habitat with gorillas were available.

We identified *E. cuniculi* genotypes I and II with genotype II as the most prevalent. The same genotypes of *E. cuniculi* have also been detected in both free-ranging western lowland gorillas and captive chimpanzees from several zoos and sanctuaries, though the *E. cuniculi* genotype I was the most prevalent one in previous studies [5], [12]. Since *E. cuniculi* genotypes are not host-specific and have been identified in a variety of animal hosts such as rodents, lagomorphs, carnivores, horses, human and nonhuman primates, and birds [12], [53], [56]–[60], detection of the exact origin of *E. cuniculi* infections in gorillas is difficult.

Surprisingly we did not detect any *E. intestinalis* in our sample set, although it is the only microsporidium species that has been previously identified in mountain gorillas and people who shared habitats with them in Uganda [2]. However, we cannot exclude that gorillas we studied do not have also concurrent *E. intestinalis* infection as diagnosis of mixed infections is limited by amplification of the predominant species over the others as it was observed in *Cryptosporidium* spp. mixed infections [61]. Additionally, our results are hampered by the one-shot sampling methodology, which could not provide precise estimation of prevalence due to intermittent shedding of infectious stages of parasites into the environment, especially in the case of *Giardia* sp. and microsporidia. A similar situation has been described in other hosts including humans [62]–[64].

We did not amplify any of *Cryptosporidium* species/genotypes previously detected in gorillas. While Graczyk et al. [33] identified zoonotic *Cryptosporidium parvum* genotype 2 in mountain gorillas, and Sak et al. [12] reported *C. bovis* in western lowland gorillas, we detected *C. meleagridis* and *C. muris* in two of examined gorillas. Typical host spectrum of *C. meleagridis* includes birds from orders Galliformes, Columbiformes, Psittaciformes and Passeriformes [65]–[67], while *C. muris* (in several genotypes) parasitizes rodents and artiodactyls [68].

No case of non-human primate infection by *C. meleagridis* has been reported so far and *C. muris*-like infection has been previously reported only in stomach of cynomolgus monkeys (*Macaca fascicularis*) [69]. However, both these cryptosporidia species are known to infect “non-typical” hosts [70], [71], being reported also from immunocompetent and immunodecificient humans. The source of cryptosporidial infection in mountain gorillas reported in this study is unknown, however, it is possible that under certain conditions this host specific cryptosporidia can be transmitted among species [70], [71], especially because both detected species has been previously reported to be human pathogenic in both immunocompetent and immunodecificient human patients. *C. meleagridis* is the third most common causative agents of human cryptosporidiosis in the world [72], whereas *C. muris* was detected only occasionally [73]–[77].

Our results imply that gorillas habituated and used for research were significantly more parasitized with *E. cuniculi* compared to gorillas habituated and used for tourism. Also *Cryptosporidium* sp. infections were found only in research groups. It might imply that research activities could pose greater risk than tourism, for example animals from research groups might be more stressed (and thus immunosuppressed) because perhaps people spend longer period with them (four hours per day) in comparison to tourist ones (one hour per day), but preliminary studies did not show any difference in fecal cortisol levels between. Noteworthy, Pablo and Shinda (as well as Susa, which is a tourist group) ranged nearby the southern edge of the Park, where more illegal activities occur [78]. This can also lead to increased stress and possibly increased risk of pathogen transmissions due to more frequent uncontrolled human presence in gorilla habitats. A closer look to our results revealed that these three groups are infected by *E. cuniculi* II while the others harbor genotype I; also the novel genotypes of *E. bieneusi* occurred only in Susa, Pablo and Shinda (Table 1). The overlapping habitat also raises the question of other animals (e.g. buffalo or elephants) or water sources, which can serve as reservoirs, but there are no apparent differences between the areas where these groups range and the rest of the Park (Cranfield, pers. obs.).

Our data showed the presence and diversity of important opportunistic protists in Volcanoes gorillas. However, the source, the routes of the circulation and the importance of these pathogens for health of gorillas remain in mist. Answering the persisting questions apparently requires bigger effort, involving: (i) broad sampling of other hosts sharing the habitat with gorillas, (ii) repeated sampling of individuals and (iii) quantification of studied protists and associating the results with actual health parameters of sampled individuals. Having all these data together is crucial for development of well-managed ecotourism and research activities with minimal impact on the animal health.
